# Pre-Season Nutritional Intake and Prevalence of Low Energy Availability in NCAA Division III Collegiate Swimmers

**DOI:** 10.3390/nu15132827

**Published:** 2023-06-21

**Authors:** Dylan J. Klein, Patrick McClain, Victoria Montemorano, Alaina Santacroce

**Affiliations:** Department of Health and Exercise Science, Rowan University, Glassboro, NJ 08028, USA

**Keywords:** energy availability, relative energy deficiency in sport, sport nutrition, collegiate swimmers, LEAF-Q

## Abstract

There is limited information regarding the dietary habits and energy availability (EA) of collegiate athletes. Therefore, the purpose of the present study is to assess the nutrient intakes, dietary habits, and prevalence of low EA (<30 kcals/kg FFM) in a group of National Collegiate Athletic Association (NCAA) Division III male and female swimmers. Energy and nutrient intake, body composition, and exercise energy expenditure was assessed in 30 (*n* = 15 males, *n* = 15 females) NCAA Division III swimmers during pre-season using three-day diet and seven-day activity records alongside multi-frequency, bioelectric impedance analysis. A validated screening tool was used to assess for low EA in the female swimmers. Mean EA in male and female athletes was 32.7 ± 12 and 34.9 ± 13.7 kcals/kg FFM, respectively, and was not significantly different between the sexes (*p* = 0.65). Twenty percent of swimmers (*n* = 3 males, *n* = 3 females) presented with optimal EA, 37% (*n* = 5 males, *n* = 6 females) presented with sub-optimal EA, and 43% (*n* = 7 males, *n* = 6 females) presented with low EA. Swimmers who presented with a low EA consumed significantly less calories, carbohydrates, and proteins than non-low EA swimmers (*p* < 0.02). The validated screening tool failed to classify 50% of female swimmers who presented with low EA. Only eight athletes achieved the USDA MyPlate recommendation for fruits, whereas three athletes achieved the recommendation for vegetables, with no differences between the sexes (*p* > 0.05). The present findings show that there was a high prevalence of low EA during the pre-season among male and female collegiate swimmers that was not fully captured using a validated screening tool for females. Low EA occurred alongside lower intakes of calories, carbohydrates, and proteins, and the majority of swimmers did not meet the United States Department of Agriculture recommendations for fruit and vegetable intake. These data stress the need for improved dietary intakes in NCAA Division III collegiate swimmers.

## 1. Introduction

Sport performance can be optimized when dietary intake meets the nutritional demands of training and competition [1]. Collegiate athletes often fail to meet dietary requirements [2,3,4] likely due to their increased need for energy and macronutrients [5] in the face of rigorous training, travel, and academic schedules. The pre-season timeframe in particular represents an additional barrier to collegiate athletes as it is characterized by the transition from lower to higher training volumes in the effort to get athletes in competition shape as quickly as possible. As a result, insufficient energy intake (EI) predisposes these athletes to low energy availability (EA). Energy availability is defined as the amount of dietary energy available to support the cost of physiological function (e.g., reproductive function) after accounting for the energetic cost of exercise [6]. Mathematically, this amounts to the difference between EI and exercise energy expenditure (EEE), normalized to fat-free mass (FFM): *EA = (EI − EEE)/FFM*. Sustained, clinically low EA (i.e., <30 kcals/kg FFM), either through reduced EI or excessive EEE, can result in disrupted metabolic and endocrine function that leads to poor reproductive, bone, and cardiovascular health [7]. Further, this can potentially lead to reduced performance and increased risk of injury [8]. Together, these factors coalesce into a condition termed Relative Energy Deficiency in Sport (RED-S) [9] and pose a major concern for athletes and coaches seeking to optimize sports performance and wellbeing.

Several investigations have assessed EA in athletic populations, primarily in endurance sports such as distance running [2] and soccer [3] where EEE is highest as is the risk for developing clinically low EA. Given the well-defined negative outcomes that accompany low EA as described as part of the Female Athlete Triad (TRIAD) [6], female athletes have garnered the majority of research attention. However, investigations have started to explore low EA in male athletes as well [10]. To date, it is unclear whether male endurance athletes exhibit a similar prevalence of low EA relative to their female peers. Recent evidence suggests this might be the case [2]; however, more work is greatly needed.

Collegiate swimmers represent an athletic population at risk for not meeting sport nutrition recommendations and developing low EA given their high training loads and the need for a leaner physique, all coupled with other scholastic requirements and university-related commitments that pose barriers to optimal nutrition. Furthermore, while National Collegiate Athletic Association (NCAA) Division III athletes comprise the largest proportion (40%) of NCAA athletics [11], they receive the least in terms of institutionally backed nutrition resources (e.g., training tables and team-dedicated dietitians). Taken together, this places NCAA Division III swimmers at particular risk for poor nutritional intake, developing low EA, and experiencing the negative consequences of RED-S.

Given the dearth of studies regarding the dietary habits and EA of collegiate athletes, particularly male athletes and swimmers in general, the aim of the present study was to assess the nutrient intakes, dietary habits, and prevalence of low EA in a group of NCAA Division III male and female swimmers. Additionally, we sought to determine the relationship between EA and various body composition variables that are important for athletic prowess.

## 2. Materials and Methods

### 2.1. Participants

The Rowan University Institutional Review Board (IRB) approved this study. Fifteen male and fifteen female NCAA Division III swimmers voluntarily agreed to participate in this study by providing written informed consent. Data were collected prior to the start of the regular swim season. Athlete characteristics are located in Table 1.

### 2.2. Energy Intake

We estimated the EI of participants using a 3-day diet record. We instructed each athlete on how to keep a 3-day diet record and provided them with a detailed instruction manual for them to reference. Participants recorded their usual diets, including supplement usage, over the course of one week by providing dietary information from two, non-consecutive weekdays and one weekend day. Upon return, we reviewed each diet record with the participant for completeness and accuracy. Additional information (e.g., brand names, volume/size measurements, and cooking methods) was added to the diet records when appropriate. We analysed energy (kcals/d), macronutrient intake (g/d), and fruit and vegetable consumption (cups/d) using ESHA Food Processor (Salem, OR) professional dietary software. Relative energy and macronutrient intakes were normalized to body mass (g/kg/d).

### 2.3. Exercise Energy Expenditure

We estimated EEE using a 7-day exercise record for all participants. Exercise records included all structured physical activity conducted, including modality, duration, and intensity. Exercise records were then compared to The Compendium of Physical Activities to calculate metabolic equivalents (METs) for each type of exercise activity. We used the following equation to calculate EEE:

EEE = body mass (kg) × time (h) × METs (1 kcal/kg/h)

### 2.4. Body Composition

We assessed body composition (body mass (BM), fat mass (FM), fat-free mass (FFM), percent body fat (%BF), and skeletal muscle mass (SMM)) using the InBody 770 multi-frequency bioelectric impedance analysis (BIA) unit (Cerritos, CA, USA). All measurements were conducted between 7:00 and 10:00 a.m. in a fasted state (i.e., no food 8–9 h prior to testing).

### 2.5. Energy Availability

Using EI, EEE, and FFM measurements, we calculated EA using the following equation:

EA = (energy intake [EI] − exercise energy expenditure [EEE])/fat-free mass (FFM)

Energy availability categories were defined as follows: optimal EA (≥45 kcals/kg FFM/d), sub-optimal EA (30–44 kcals/kg FFM/d), and clinically low EA (<30 kcals/kg/FFM/d) [12].

### 2.6. Low Energy Availability in Females: Questionnaire

To screen for female athletes as having low EA, and thus at risk for TRIAD/RED-S, we used the low energy availability in females questionnaire (LEAF-Q). The 25-item questionnaire was given to each female swimmer. It asked a series of questions related to prior injury, gastrointestinal issues, menstrual cycle function, and contraceptive use. A score ≥ 8 classified the athlete as having low EA. The LEAF-Q has a reported sensitivity of 78% and specificity of 90% with a Cronbach’s alpha ≥ 0.71 [13].

### 2.7. Statistical Analysis

Participant demographic information is presented using descriptive statistics (means ± standard deviations (SD)). Differences between the sexes and between athletes with and without clinically low EA were carried out using Student’s *t*-test. Cohen’s d effect sizes were calculated for energy intake variables between athletes with and without clinically low EA. Effect sizes were interpreted using the following criteria: <0.2 = trivial; 0.2–0.6 = small; 0.7–1.2 = moderate; 1.3–2.0 = large; >2.0 = very large. Pearson correlation coefficients were used to examine the relationships between EA and body composition variables. Correlations were interpreted using the following criteria: very weak: <0.20; weak: 0.20–0.39; moderate: 0.40–0.59; strong: 0.60–0.79; very strong: >0.80 [14]. Macronutrient intake values were compared to the most recent sport-recommended intakes (SRI) set forth by the American College of Sports Medicine, the Academy of Nutrition and Dietetics, and the Dietitians of Canada Joint Statement on Nutrition and Athletic Performance [15]. Fruit and vegetable intake was compared to the most recent United States Department of Agriculture (USDA) MyPlate recommendations established as part of the Dietary Guidelines for Americans, 2020–2025 [16]. All statistical analyses were conducted using GraphPad Prism software version 9.5.1 for Windows (San Diego, CA, USA). Significance for all tests was set at an alpha of ≤0.05.

## 3. Results

### 3.1. Participants

Athlete characteristics are located in Table 1. Male and female swimmers were similar in age and BMI (*p* > 0.05); however, males were significantly different from females regarding height, BM, FM, FFM, %BF, and SMM (*p* < 0.0002).

### 3.2. Energy Intake, Exercise Energy Expenditure, and Macronutrient Intake by Sex

**Figure 1:**
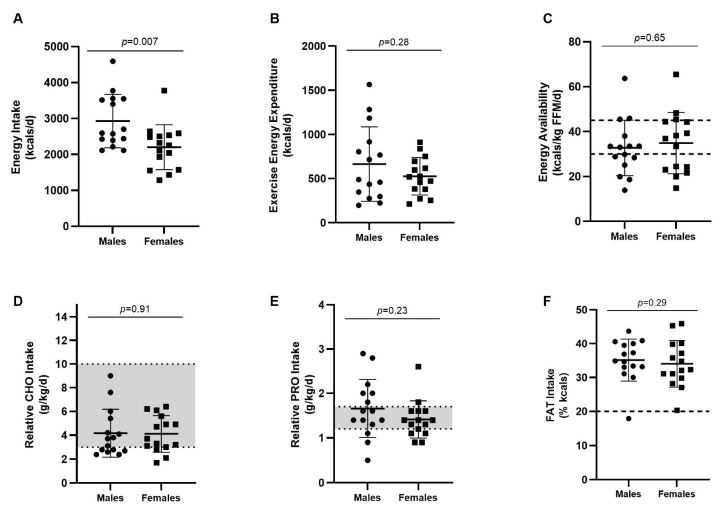
Energy intake, exercise energy expenditure, energy availability, and macronutrient intakes in male and female collegiate swimmers. Circles indicate males (*n* = 15), squares indicate females (*n* = 15). Values are reported as means ± SD (**A**–**F**). Optimal (≥45 kcals/kg FFM), sub-optimal (30–44 kcals/kg FFM), and clinically low (<30 kcals/kg FFM) EA cut-offs are indicated by dashed lines (**C**). Grey shaded areas (**D**,**E**) represent SRI ranges for CHO (3–10 g/kg/d) and PRO (1.2–1.7 g/kg/d). The SRI threshold for FAT (≥20% kcals) is represented by a dashed line (**F**). *p* = 0.007, energy intake significantly differed between males and females based on Student’s *t*-test. CHO = carbohydrate; FAT = fat; FFM = fat-free mass; PRO = protein.

Athlete EI, EEE, and relative macronutrient intakes are located in Figure 1. Males consumed significantly more kcals per day than females (2930.4 ± 717.8 vs. 2201.8 ± 625.2 kcals/d, respectively; *p* = 0.007) but were similar in their EEE (664.2 ± 406.7 vs. 525.6 ± 211.2 kcals/d, respectively; *p* > 0.05). Males consumed significantly more protein (130.2 ± 48.3 vs. 91.5 ± 25.0 g/d, respectively; *p* = 0.01) and fat (113.7 ± 34.5 vs. 81.7 ± 22.9 g/d, respectively; *p* = 0.005) than females, but were not significantly different in their absolute intake of carbohydrates (323.8 ± 134.8 vs. 261.0 ± 71.9 g/d; *p* = 0.13). Relative EIs of carbohydrates (4.2 ± 2.0 vs. 4.1 ± 1.5 g/kg), proteins (1.7 ± 0.7 vs. 1.4 ± 0.4 g/kg), and fats (1.4 ± 0.5 vs. 1.3 ± 0.4 g/kg) were not significantly different between males and females, respectively (*p* > 0.05, Figure 1).

Forty percent of males (*n* = 6) and 20% of females (*n* = 3) did not meet the SRI for carbohydrates (i.e., 3–10 g/kg/d), whereas 33% of both males (*n* = 5) and females (*n* = 5) did not meet the SRI for protein (i.e., 1.2–1.7 g/kg/d). All but one athlete (male) consumed the SRI for dietary fat (i.e., >20% kcals).

Fifty-three percent of males (*n* = 8) and 40% of females (*n* = 6) reported supplement usage. The most commonly consumed supplements were protein powder/bars (five males, four females), multi-vitamins (one male, three females), and energy bars (two males, one female). One male also reported taking a pre-workout supplement containing caffeine.

### 3.3. Energy Availability

Mean EA in male and female athletes was 32.7 ± 12 and 34.9 ± 13.7 kcals/kg FFM, respectively, and was not significantly different between the sexes (*p* = 0.65, Figure 1). Using established cut-offs for optimal (>45 kcals/kg FFM/d), sub-optimal (30–44 kcals/kg FFM/d), and clinically low EA (<30 kcals/kg/FFM/d), 20% of swimmers (*n* = 3 males, *n* = 3 females) presented with optimal EA, 37% (*n* = 5 males, *n* = 6 females) presented with sub-optimal EA, and 43% (*n* = 7 males, *n* = 6 females) presented with clinically low EA. Differences in EA and macronutrient intake between swimmers with and without low EA can be found in Table 2.

In the female swimmers, the LEAF-Q questionnaire was also used to screen for low EA. Of the six female athletes who presented with clinically low EA, the LEAF-Q accurately identified three (50%). Additionally, the LEAF-Q categorized three female swimmers as having clinically low EA but who did not present with low EA based on diet and body composition analyses (i.e., all had EA values > 38 kcals/kg FFM).

### 3.4. Correlational Analyses

Correlational analyses between EA and body composition parameters can be found in Table 3. Of note, a significant, moderate inverse correlation was found between EA and BM (*p* < 0.02).

### 3.5. Fruit and Vegetable Intake

**Figure 2:**
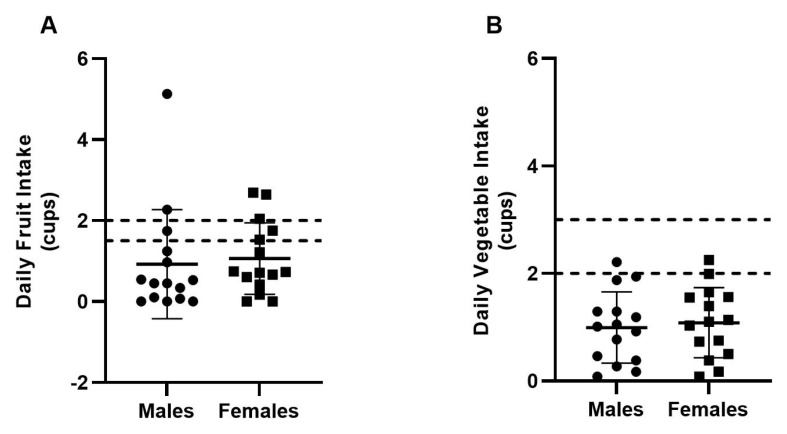
Daily fruit and vegetable intake in male and female collegiate swimmers. Circles indicate males (*n* = 15), squares indicate females (*n* = 15). Values are reported as means ± SD. USDA MyPlate recommended intakes for fruits (1.5–2 cups/d) and vegetables (2–3 cups/d) are indicated by dashed lines (**A**,**B**).

We assessed fruit and vegetable intake in the present study (Figure 2). Only eight athletes (*n* = 3 males, *n* = 5 females) achieved the USDA MyPlate recommended intake for fruits (i.e., 1.5–2 cups/d), whereas three athletes (*n* = 1 male, *n* = 2 females) achieved the recommended intake for vegetables (i.e., 2–3 cups/d). There were no differences between the sexes (*p* > 0.05).

## 4. Discussion

The purpose of the present study was to assess the nutrient intakes, dietary habits, and prevalence of clinically low EA during the pre-season in a cohort of NCAA Division III collegiate swimmers. To the best of our knowledge, this is the first investigation of this kind in collegiate-level swimmers, and it adds to the growing literature on EA in male athletes. The primary findings of the study show the mean EA in male and female athletes was 32.7 ± 12 and 34.9 ± 13.7 kcals/kg FFM, respectively, and the prevalence of clinically low EA to be 43% with no significant differences between male (47%) and female (40%) swimmers. The prevalence of clinically low EA reported in the present study is higher than some published findings in collegiate-track [17] and soccer [18] athletes; however, these results fall within the overall reported range of 20–67% for clinically low EA amongst collegiate and elite endurance athletes [2,3,17,18,19,20]. Similarly, our findings concur with a recent publication by Beermann et al. [2] who showed 75–80% prevalence of sub-optimal EA and no difference in the prevalence of low EA between male and female collegiate distance runners (i.e., 45% and 41%, respectively). While these results suggest that male athletes exhibit a similar prevalence of clinically low EA compared to their female counterparts, it is worth noting that there is no consensus on what constitutes clinically low EA in males [21]. As such, most studies on male athletes rely on a cut-off of 30 kcals/kg FFM that may not be sufficient to induce the metabolic and endocrinologic consequences of low EA seen in females [22,23]. More research is therefore needed to establish a male-specific cut-off for clinically low EA [21] to better compare prevalence across sexes and within sporting disciplines.

Unsurprisingly, swimmers with clinically low EA consumed less kcals, carbohydrates, protein, and fats compared to swimmers without clinically low EA. This held true for both absolute (g/d) and relative (g/kg/d) intakes. As a consequence, these athletes failed to meet the SRI threshold for carbohydrates (i.e., 3 g/kg) but did meet the lower end of the SRI threshold for protein (i.e., 1.2 g/kg). Previous research shows that consuming fewer carbohydrates is observed more commonly in athletes who present with clinically low EA [18,24,25,26,27], whereas it is unclear whether athletes who have clinically low EA consume less [28], more [26], or the same [3] amount of protein (on a g/kg basis) as their energy-sufficient counterparts. Further, while these measurements are only reflective of the pre-season timepoint, the potential inability to compensate for these intakes during the regular season could negatively affect the health and performance of these athletes as mediated through losses of glycogen stores, lean body mass, and power/strength [29]. Indeed, Vanheest and colleagues demonstrated a 9.8% decrease in 400 m swim velocity in junior elite female swimmers who exhibited clinically low EA during a competitive season [30].

Early identification of low EA can help inform interventions that help athletes during the regular season attain optimal EA and macronutrient intakes. In this vein, the LEAF-Q instrument was used to assess for low EA and thus the risk for developing TRIAD/RED-S in the female swimmers. Similar to other recent publications [3,31], the LEAF-Q was not effective in discriminating between female swimmers with and without clinically low EA. With regard to the male swimmers, at the time of the study, no screening tool was present to assess for low EA. While recent studies have endeavoured to validate such a screening tool [32], little success has been made, which reinforces the need for low-burden discriminators of male athletes with low EA.

Interestingly, the current investigation showed a moderate significant inverse association between BM and EA. Indeed, non-low EA athletes were lighter than their clinically low EA counterparts, and this trended toward significance (*p* = 0.059). This is in agreement with a recent paper by Magee et al. who also showed a moderate significant inverse relationship between BM and EA in NCAA Division III female soccer athletes [3]. This finding suggests that larger collegiate athletes may have difficulties meeting their energy requirements as a function of their body size that requires higher energy needs in the face of arduous training, competition, and academic schedules. Coaches and nutritionists could use this information to better identify and monitor athletes at risk for developing low EA and RED-S during the season and before negative performance and health consequences take place.

Inadequate intake of fruits and vegetables places athletes at risk of under-consuming key micronutrients and antioxidants that promote health and bodily function [5]. We discovered that only 27% of swimmers in the present study consumed the USDA recommendation for daily intake of fruits and a paltry 10% consumed the recommendation for vegetables. Very few studies have assessed the fruit and vegetable intake of collegiate athletes [33,34], thus making comparisons in the literature difficult. A recent qualitative study by Eck and Byrd-Bredbenner [35] revealed that Division I athletes (*n* = 14, 64% female) commonly reported eating fruits and vegetables “every day or almost every day”. This, however, contrasts our results as well as a study by Abbey et al. [34] who showed that fewer than 50% of Division III football players (*n* = 88 in total) consumed fruits and vegetables daily. Taken together, it appears that achieving optimal intakes of fruits and vegetables, as per USDA guidelines, is an issue in collegiate athletes that needs addressing.

### Study Limitations

This study is not without limitations. As with any investigation of free-living dietary intake, self-reported measures are subject to mis/underreporting [36]. While efforts were taken to minimize these negative aspects, the potential for overestimating the prevalence of low EA cannot be ignored. Similarly, accurate accounting of all physical activity is limited by the self-reported nature of the data. A recent opinion publication by Taguchi and Manore [37] highlighted the need for better intake measurements of EEE. In that paper, the authors recommended accounting for all physical activity, not just structured physical activity, to better estimate EA. Future studies using accelerometers and heart rate monitors could be useful to better account for additional sources of physical activity that participants often failed to record.

Another limitation of the current study is the use of BIA to estimate FFM. Previous research has shown that BIA tends to overestimate FFM in athletic populations [38,39], thus potentially overestimating low EA in our cohort. Using a more objective measure such as RMR_ratio_ (i.e., the ratio of resting metabolic rate measured divided by the predicted rate) could help to solve this issue, as well as underreporting, by directly assessing an individuals’ metabolism that is indicative of EA status. Indeed, an RMR_ratio_ < 0.90 has been suggested to be representative of a low EA status [40,41]. Finally, the study is hindered by its relatively small sample size. Therefore, these findings may not be representative of all swimmers across all levels of NCAA competition.

## 5. Conclusions

In conclusion, the results of the present study indicate that there may be a high prevalence of clinically low EA among male and female NCAA Division III swimmers, and that it is more common in swimmers with greater BM. We also demonstrated a fair number of swimmers who failed to meet the SRI for carbohydrates and protein. Further, the vast majority of swimmers failed to consume the USDA MyPlate recommended daily intake for fruits and vegetables, indicating the poor diet quality of these athletes and the risk for under-consuming key nutrients that regulate health and function. Lastly, the LEAF-Q questionnaire was not a reliable instrument in identifying female athletes with clinically low EA, and future studies should aim to find a more appropriate tool, not only for females but for male athletes as well.

## Figures and Tables

**Table 1:** Athlete characteristics.

Characteristic	Males (*n* = 15)	Females (*n* = 15)
Age (yrs.)	19.9 ± 1.2	19.7 ± 1.5
Height (cm)	183.7 ± 7.0	168.3 ± 6.0 *
Body mass (kg)	79.5 ± 8.7	65.8 ± 8.5 *
BMI (kg/m^2^)	23.6 ± 2.3	23.2 ± 2.5
FM (kg)	9.3 ± 3.3	16.9 ± 5.4 *
%BF	11.1 ± 3.6	25.3 ± 5.6 *
FFM (kg)	70.2 ± 7.0	48.8 ± 5.0 *
SMM (kg)	40.3 ± 4.2	27.1 ± 3.0 *

Data presented as means ± SD. %BF = percent body fat; BMI = body mass index; FFM = fat-free mass; FM = fat mass; SMM = skeletal muscle mass. * *p* < 0.0002, significantly different from males based on Student’s *t*-test.

**Table 2:** Daily energy and macronutrient intake between swimmers with and without low EA.

	Low EA (*n* = 13)	Non-Low EA (*n* = 17)	Effect Size
BM (kg)	77 ± 9.4	69.3 ± 11.4 ^^^	0.7
Energy availability (kcals/kg FFM)	22.5 ± 5.1	42.5 ± 9.8 ****	1.5
Energy intake (kcals/d)	2007.1 ± 423.6	2933.5 ± 701.0 ***	1.4
Relative energy intake (kcals/kg/d)	26.0 ± 4.2	43.5 ± 8.9 ****	2.0
Carbohydrate intake (g/d)	212.3 ± 43.6	353.7 ± 113.1 ***	1.3
Relative carbohydrate intake (g/kg/d)	2.8 ± 0.5	5.2 ± 1.6 ****	1.5
Protein intake (g/d)	90.6 ± 31.0	126.4 ± 44.4 *	0.8
Relative protein intake (g/kg/d)	1.2 ± 0.3	1.8 ± 0.5 ***	1.2
Fat intake (g/d)	79.8 ± 20.7	111.5 ± 34.5 **	0.9
Relative fat intake (g/kg/d)	1.0 ± 0.2	1.6 ± 0.4 ***	1.4
Fat intake (% kcals)	35.7 ± 5.0	33.7 ± 7.4	0.3

BM = body mass; FFM = fat-free mass; EA = energy availability. * *p* < 0.02, ** *p* < 0.01, *** *p* < 0.001, **** *p* < 0.0000, significantly different from low EA based on Student’s *t*-test. ^^^ *p* = 0.059, trend toward significant difference from low EA based on Student’s *t*-test.

**Table 3:** Relationships between body composition and energy availability in swimmers.

	Mean EA (kcals/kg FFM)
**BM (kg)**	−0.424 *
**%BF**	−0.057
**FFM (kg)**	−0.271
**FM (kg)**	−0.221
**SMM (kg)**	−0.266

%BF = percent body fat; BM = body mass; EA = energy availability; FFM = fat-free mass; FM = fat mass; SMM = skeletal muscle mass. * *p* < 0.02 based on Pearson correlational analysis. Very weak: <0.20; weak: 0.02–0.39; moderate: 0.40–0.59; strong: 0.60–0.79; very strong: >0.80.

## Data Availability

Data pertaining to this study can be made available upon request.
